# Supplementary material for: “My Health Is More Important than Drinking”: A Qualitative Analysis of Alcohol Use During COVID-19
Source: Int J Environ Res Public Health. 2025 Feb 5;22(2):224. doi: 10.3390/ijerph22020224 (PMC11855319; doi:10.3390/ijerph22020224)
Supplement: Supplementary file 1 [file ijerph-22-00224-s001.zip › ijerph-3412852-supplementary.pdf]

Supplementary Table S1. Summary of identified themes and exemplar quotes.

Theme	Exemplar Quotes
Concerns about alcohol negatively affecting health	I probably would say that it was the surgery and recovering from the surgery. Because I think under normal circumstances [my alcohol use] would have been the same (Participant 1, 44-year-old Hispanic Black Woman).<br><br>Actually, I think I stopped drinking in March because I was on all of these medicines and I was worried about the interactions or the infection (Participant 22, 25-year-old non-Hispanic White Woman).<br><br>Because like I said, I already suffer from depression, but this just adds, you know, even more to it. It exacerbates all my symptoms. It just, it's just not helpful at all. But and it's something I keep thinking about, because it's like, you know, it's taking up a lot of time because I'm like, I don't want to be addicted to alcohol. I don't want to depend on alcohol. (Participant 10, 40-year-old non-Hispanic Black Woman)<br><br>Now, well, the thing is I really don't have an option. I can't be drinking. Like, I would want to drink a beer, but I really can't drink. I guess my health is more important than drinking. So, I'm going to stick to the same routine. I'm not drinking that much. (Participant 12, 54-year-old non-Hispanic White Man)<br><br>It was very new, but what I will say is that I do think-- I do notice a difference now that I haven't been drinking as much because of my doctor's orders for me to stop drinking. I feel a lot less anxious now but I also didn't feel, when I was drinking as much, that I had a-- when you see people and you say, "I think you may have a problem and maybe need to talk to somebody about your habits and other ways, and better ways to cope," I think my doctor had the conversation with me kind of really indirectly in that way and because it was tied to a medical issue, I was able to stop it almost immediately. So, I do feel better about me being bale to remove myself from the habit and mental health wise, it makes me feel better as well. I'm

	not as anxious anymore even though work is so stressful. I do more productive things though as a result. (Participant 7, 36-year-old non-Hispanic Black Man)<br><br>So and I know kind of it's at the end of November, but I primarily noticed at the beginning of November, my liver would start hurting periodically. And I was like, "Yeah, that's probably from all the drinking and nicotine I've been smoking." So I decided I should stop drinking. I just cut back on drinking. So I think it was, like, the 14th or I think it was, like, the 14th of December, maybe 15th, decided I'm not going to drink anymore until the beginning of the year. (Participant 8, 27-year-old non-Hispanic American Indian/Alaskan Native Man)
Reduce chance of experiencing drinking problems faced by loved ones	I've seen, through my family, how alcohol has really negatively impacted people. They drink instead of going out to work. They'll drink in the morning before going to work to kind of help them get their day started and I've seen the negative effects of liver damage on family members and things like that as well…and I never wanted to see myself like that. (Participant 7, 36-year-old non-Hispanic Black Man)<br><br>I know that there's other ways to cope... I know that my grandmother was an alcoholic, while my dad was an alcoholic and- and a drug user. But I don't want to be like them. But it's the hardest thing. (Participant 10, 40-year-old non-Hispanic Black Woman)<br><br>Interviewer: Your drinking has been pretty consistent. How do you understand that?<br>Participant: I grew up, my dad is a very, very good person but he's been one to have a very, I don't know the word in English. He's besedoso, [ph?] he kind of affixes to things and then he was smoking. He gave up smoking back in 2003 because he had a very traumatic experience where he had to get rushed to the hospital and his heart, he had a valve that just shut and he dropped it overnight. It was nothing short of miraculous. It was like, okay. He had been smoking for forty years, two packs of cigarettes a day, I'm not kidding, and he just dropped it.<br>Interviewer: That's impressive.<br>Participant: I mean, when he stops, he stops. Then <speaks Spanish> he picked up drinking and he was getting very drunk, very excessively drunk, every day for another, I don't know, probably another ten years. Well, in 2016 was when he

	stopped because it was right, the day of my baby shower with my first son, he had another-- his nose turned blue and his oxygen-- his face was turning purple and his oxygen was just dropping. This was a great ending to my first baby shower. I was like, “What is going on?” It was wild. So, I think just having had those experiences myself, I enjoy drinking as much as anybody. You can ask anybody. I will take a shot in celebration of something. <speaks Spanish> I do, and I don’t have any sort of weird signal. I mean, I don’t think it’s good to get drunk but there’s just physical and emotional repercussions that come with it. I don’t think it’s a smart idea, but I don’t think it’s the worst thing in the world, but I think having grown up with that and dealing with that, I just, I’m like, “Okay, I’m feeling a little tipsy or I’m feeling a little buzzed, that’s it, I’m done. I don’t need anymore.” I think it just has a lot to do with that, honestly. (Participant 24, 27-year-old Hispanic White & Middle-Eastern Woman)
Life demands and transitions limiting drinking opportunities	…it feels like non-stop busyness. I'm drinking less because there's so many things going on. And also we're trying to save money for this wedding because it's already like getting very expensive. It's most stressful right now. I'm like the busiest right now, but like it's not an emotional stressor. I feel very happy. Yeah. But I'm very busy... (Participant 26, 24-year-old non-Hispanic White Woman)<br><br>Well, I'm kind of consistent consistently. You know, I have a routine. I maintain a routine. So, it’s like I do that, you know, not going out as much and going out to places just staying home. But staying home hasn't caused me to drink more like maybe some people that have more time to do that they would drink more. But probably the consistency. I'm pretty consistent in, you know, my daily life, the rituals and that. (Participant 15, 65-year-old Hispanic White Man)<br><br>…now I have to leave the house to work. So that prevents me from drinking because when I was stuck here at the house, I would be drinking from the house. Now I really don't think about it. I don't really think about that bad habit. (Participant 25, 33-year-old Hispanic White Woman)<br><br>Just stayed busy. I guess it's almost like dealing with a sick loved one or somebody has-- maybe a close relative just passed away, you kind of just keep busy doing something, you keep <inaudible 00:35:28> off of it and then you're like,

	"Oh, it's ten o'clock already, it's time to go to bed." Worked the whole day or whatever. So keeping a busy schedule helps the most for me just to keep my mind off things. And that actually keeps-- I drink less if I've got a project going. I've pretty much got something going on all the time, working on the car or fixing something on the house. Even just yesterday I was working at my mom's house, like I said, they live right next door to us, so I was working on a project over there where they had a floor rotted out, so I was stripping it out and putting new floor joists and stuff like-- anyway the point is if I stay busy it's less stressful and I don't think about drinking as much. (Participant 23, 40-year-old non-Hispanic White Man)<br><br>Yeah. Well, that's the thing like when I'm most relaxed I'm drinking more because I don't have a lot on my plate. If I don't have any plans on Sunday all day I can drink what I want on Saturday and if I feel crummy on Sunday, it's fine. But like right now it's so busy that like I don't really have time to have like, a feeling crappy day. But like I really genuinely feel happier now. I know that sounds so dumb, but I like being busy. I like that it stops me from drinking a little bit because my body feels a little bit healthier. And I think, again, like my career just like took a huge like upswing. My relationship is like we're on like a huge upswing. I'm happier even though I'm busier and drinking less. But I was most relaxed when I was drinking more (Participant 26, 24-year-old non-Hispanic White Woman)
Avoiding negative side effects and after effects of drinking	When I was drinking...liquor, I guess I probably was getting <laughs> hung over a little bit and then I was like, 'Oh, I'm not going to drink for the week' (Participant 11, 36-year-old Hispanic White Woman).<br><br>If I get drunk every night for four nights in a row, I start to really feel it because I'm getting older. But it starts to drag down my normal day, so I have to quit, I just can't-- if it gets to that point I kind of start feeling it too much and my work starts to suffer and yeah, so it's always a concern. (Participant 23, 40-year-old non Hispanic White Man)<br><br>Participant: Well I always kind of have it in the back of mind that it's unhealthy and I know that it could potentially cause some damage and long term health

effects. Because if I get drunk every night for four nights in a row, I start to really feel it because I'm getting older. But it starts to drag down my normal day, so I have to quit, I just can't-- if it gets to that point I kind of start feeling it too much and my work starts to suffer and yeah, so it's always a concern, <inaudible 00:15:37> pretty steady since I guess my 20s, so that's quite a while. And even the doctor's like, "Well, you know, you're on the edge of, you're not excessive, but heavy," said I was a heavy drinker, but over a period of time that's what you got to really worry about I think is what the doctor was trying to tell me. (Participant 23, 40-year-old non Hispanic White Man)

But some of these times I just need to go instead of four or five days without drinking I have to go back to going 12 or 13 just because I feel good. You know? I feel good mentally and physically when I do that. And the body's ability to process alcohol, as you get older, it starts to go down. You know? And like-- if I drink too much now it's like two or three days to recover from that. Whereas, you know, when I was young it was two or three hours in the morning. And, also, it-- like you get down a little bit, not while you're drinking, but the next day. You feel kind of sluggish. And I'm not anyone who's ever been depressed, but you just feel a little lower than you normally do. And I attribute that to having, you know, a couple more drinks the night before. So I'm very conscious of that. And I want to kind of get off that path. (Participant 19, 51-year-old non-Hispanic White Man)

Interviewer: It sounds like it was a bunch of things. It was the mix you talked about before. Also you're just checking about your body and how you help that and support your health, and then you started going to the gym with a friend, and you were drinking more water, and things went more in that direction. It sounds like there was a move in a positive direction for you. When you talk about it, it sounds like it felt good to do. What were kind of the benefits that were happening for you? What did you notice that you liked about that?
Participant: I feel like my skin improved, actually. I didn't expect that, but I have less-- I usually have one, two pimples a month, and now I usually don't have any. I was doing gymnastics before, so I feel my body there, and now I didn't feel like-- I'm not fat, because I don't weigh a lot, but I want to be more defined, because I saw a picture from before, and I have abs and these things, but I say okay. Now

	with the gym I am a bit-- a tendency that I see more muscle, and I feel more comfortable with myself. I don’t know, since I’m watching my food more carefully, I stop taking omeprazole for my stomach, and so I think it’s less pills; it’s better. My stomach is a big point, because I am taking pills since I was a teenager for my pain, because my cardias didn’t close properly, and the food can go up. So that is a good point that I don’t take any pills anymore. Interviewer: No, that’s great. Did you feel like drinking wasn’t contributing to those problems, or was it separate? Participant: Yeah. After the one, two drink on Friday, Saturday in the morning my stomach was awful. Interviewer: So it sounds like that also contributed some. Participant: Yeah. Now I drink three at the top of a day, and I don’t-- I prefer to do less, but if some days, okay, let’s go crazy-- but my stomach notices how much drink I have. (Participant 6, 27-year-old Hispanic White Woman)
